# Is gender dysphoria associated with increased hospital cost per stay among patients hospitalized for depression? Focus on the racial and regional variance in US hospitals

**DOI:** 10.3389/fpubh.2024.1359127

**Published:** 2024-05-23

**Authors:** Sun Jung Kim, Mar Medina, Jeong-Hui Park, Na-Eun Cho, Jongwha Chang

**Affiliations:** ^1^Department of Health Administration and Management, College of Medical Science, Soonchunhyang University, Asan, Republic of Korea; ^2^Center for Healthcare Management Science, Soonchunhyang University, Asan, Republic of Korea; ^3^School of Pharmacy, University of Texas at El Paso, El Paso, TX, United States; ^4^Department of Health Behavior, School of Public Health, Texas A&M University, College Station, TX, United States; ^5^College of Business, HongIk University, Seoul, Republic of Korea; ^6^Department of Pharmaceutical Sciences, Irma Lerma Rangel School of Pharmacy, Texas A&M University, College Station, TX, United States

**Keywords:** gender dysphoria, healthcare disparity, healthcare utilization, national inpatient sample, hospital cost per stay

## Abstract

**Introduction:**

Individuals with gender dysphoria do not identify with their sex assigned at birth and face societal and cultural challenges, leading to increased risk for depression, anxiety, and suicide. Gender dysphoria is a DSM-5 diagnosis but is not necessary for transition therapy. Additionally, individuals with gender dysphoria or who identify as gender diverse/nonconforming may experience “minority stress” from increased discrimination, leading to a greater risk for mental health problems. This study aimed to identify possible health disparities in patients hospitalized for depression with gender dysphoria across the United States. Depression was selected because patients with gender dysphoria are at an increased risk for it. Various patient and hospital-related factors are explored for their association with changes in healthcare utilization for patients hospitalized with depression.

**Methods:**

The National Inpatient Sample was used to identify nationwide patients with depression (*n* = 378,552, weighted *n* = 1,892,760) from 2016 to 2019. We then examined the characteristics of the study sample and investigated how individuals’ gender dysphoria was associated with healthcare utilization measured by hospital cost per stay. Multivariate survey regression models were used to identify predictors.

**Results:**

Among the 1,892,760 total depression inpatient samples, 14,145 (0.7%) patients had gender dysphoria (per ICD-10 codes). Over the study periods, depression inpatients with gender dysphoria increased, but total depression inpatient rates remained stable. Survey regression results suggested that gender dysphoria, minority ethnicity or race, female sex assigned at birth, older ages, and specific hospital regions were associated with higher hospital cost per stay than their reference groups. Sub-group analysis showed that the trend was similar in most racial and regional groups.

**Conclusion:**

Differences in hospital cost per stay for depression inpatients with gender dysphoria exemplify how this community has been disproportionally affected by racial and regional biases, insurance denials, and economic disadvantages. Financial concerns can stop individuals from accessing gender-affirming care and risk more significant mental health problems. Increased complexity and comorbidity are associated with hospital cost per stay and add to the cycle.

## 1 Introduction

Individuals with gender dysphoria do not identify with their sex assigned at birth, and to clarify, not all individuals with gender dysphoria are transgender, and vice versa (1). Individuals with gender dysphoria or who identify as gender diverse/nonconforming may experience “minority stress” from increased discrimination, leading to a greater risk for mental health problems (e.g., depression, anxiety, and suicide) (2, 3). While gender dysphoria is a diagnosis in the Diagnostic and Statistical Manual of Mental Disorders 5th edition (DSM-5), it should not be considered a mental health problem, and its formal diagnosis is not always required for gender transition treatment in the United States (2).

The proportion of gender dysphoria in adolescents, according to a systematic review by Thompson and colleagues has not been studied using a population-based sample and could not be estimated, but over the years, individuals with gender dysphoria are diagnosed at younger ages, and the need for gender transition services has grown (4). One study found that most transgender individuals diagnosed with gender dysphoria and seeking genital gender-affirming surgery experienced gender dysphoria by age 7 and spent 22–27 years without transitioning (5). These results are similar to another article that found that feelings of depression and suicidal ideation decreased after gender transition (6). Some estimate that about 4–6% of the US population identifies as a sexual and/or gender minority (7); a 2017 study estimated that 0.5–1.3% of children, adolescents, and adults identified as transgender (3, 8). The transgender population is growing as more individuals openly identify as transgender; the 2015 US Transgender Survey had quadrupled the number of respondents as its 2008–09 predecessor (9). The Western US has the highest percentage of adults who identify as Lesbian, Gay, Bisexual, Transgender (LGBT), followed by the Northeast, then Georgia and Texas (10).

Because individuals with gender dysphoria are at risk for mental health problems (2), addressing their psychological comorbidities, like depression or anxiety, will help support their transitioning (11–15). Individuals with gender dysphoria or transgender are at-risk for suicide and depression (6, 9, 14, 16, 17) and require specialized treatment and early diagnosis to improve their quality of life (6). Transgender adolescents (14–18 years old) are more likely to commit suicide and have a higher risk for suicidal ideation than cisgender adolescents (14); and those who are transgender or gender diverse have greater rates of depression (average age 19–20 years) (11). One study using the 2015–2020 National Survey on Drug Use and Health found that across the US, depression is most common in individuals 18–25 years old, and depression in 2020 was about 20% among US adolescents (12–17 years old) and young adults (18–25 years old) (18). The increased risk for depression in adolescents and young adults correlates with research on increased mental health problems, like suicide and depression, in patients who are transgender or gender diverse (genders outside of the binary framework) in those age ranges (11, 14, 18).

Additionally, insurance and lack of healthcare access remain historical obstacles to gender-affirming and mental health treatments in the US (9, 15). Slaughter and colleagues studied bottom/genital gender-affirming surgery rates among transgender individuals with gender dysphoria (19). Researchers found that transgender patients with gender dysphoria are more likely to have gender-affirming surgery if they have private insurance, have fewer comorbidities, and are mainly from the Western US (19). Further, such procedures typically happen at urban hospitals and, on average, cost $101,654 (19). Insurance type may play an essential role in care for individuals with gender dysphoria because of the high cost of gender-affirming surgery and medications (19, 20). Another study found a high proportion of depression among patients with gender dysphoria and low insurance coverage among those who used hormone therapy (21). Racial and ethnic minorities also tend to have lower insurance rates than White individuals (22), which could impede their access to care. Compared to other members of the LGBT community, transgender individuals are more likely to delay care and describe adverse outcomes from disclosing their transgender identity to providers (22).

Literature on individuals with gender dysphoria is limited (4, 15, 17, 19, 23), and more research is required to identify health disparities by race. Current research has found that differences in diagnosis, comorbidities, and health outcomes exist by race (24–28). For example, racial and ethnic minorities are less likely to be diagnosed with gender dysphoria or receive hormonal treatment than non-Hispanic White individuals and more likely to be refused care (24, 29). Multiple studies have also found racial differences in surgical outcomes from gender-affirming surgeries, with Black individuals more likely to need reoperation or have complications (26–28). Understanding how patients with gender dysphoria access healthcare can illuminate strategies to improve access and target preventative mental health treatment.

Research on individuals with gender dysphoria is expanding, but more significant work is required to capture current obstacles and improve mental health outcomes. Gender-affirming and depression therapies are costly (19, 30). While not all individuals with gender dysphoria are interested in gender-affirming care, past research has shown that gender-affirming treatments, for those who wish them, reduce depressive symptoms (6, 31–33). Hospitalization for depression can cost thousands of dollars and commonly has readmissions (30). Therefore, individuals with depression and who are seeking gender-affirming care could be deterred by current and future hospital costs.

This study aims to explore differences in hospital cost per stay for individuals with gender dysphoria and depression by patient and hospital factors. Patient factors include demographics like race, ethnicity, income, and insurance type. Hospital factors are hospital location, type, and size. The goal of the study is to bring attention to the financial struggles that patients with gender dysphoria and depression may face to bring support to this community and promote mental wellness. By exploring differences in cost for patients already struggling with mental health, this study can identify populations of greater need and disproportionate burdens that could prevent them from seeking further care.

## 2 Materials and method

### 2.1 Data collection

**Figure 1:**
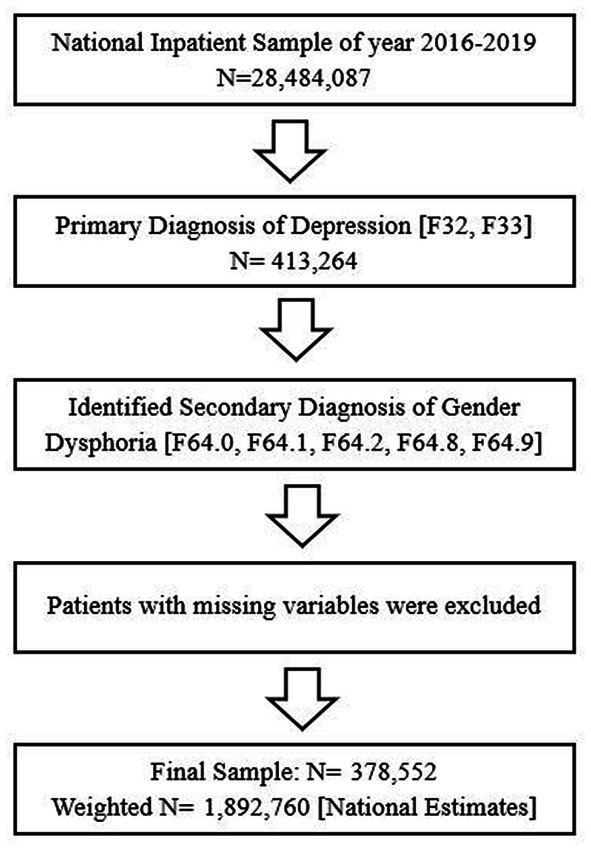
Flow chart of patient sample selection.

The 2016–2019 National Inpatient Sample (NIS) database was used to obtain a population-based estimate for nationwide patients with depression (F32, F33). With over 7 million hospitalizations annually, roughly 20% of all inpatient discharges nationwide, the NIS is the largest publicly accessible all-payer inpatient database in the country. Using the weight, we are able to measure nationwide estimates. First, among all 2016–2019 NIS samples (*N* = 28,484,087), shown in Figure 1, we identified a primary diagnosis of depression (*n* = 413,264) using the International Classification of Diseases Tenth Revision (ICD-10-CM/PCS) codes for major depressive disorder (34). Then, we identified a secondary diagnosis of gender dysphoria using the ICD-10 codes for gender dysphoria (F64.0, F64.1, F64.2, F64.8, F64.9) (34) among those with depression. Finally, after missing variables were excluded, we obtained final depression inpatients (*N* = 378,552 Weighted *N* = 1,892,760) with/without gender dysphoria (With: 14,145/Without: 1,878,615) for final analysis. The data we use is secondary data, and all the patients’ information is encrypted and unable to identify. This study was approved for a waiver from the Institutional Review Board, Soonchunhyang University (202203-SB-027).

### 2.2 Variables

The primary outcome was to investigate the association between gender dysphoria and hospital cost per stay among patients with depression. Therefore, we included the hospital cost per stay variable in the dataset as the primary outcome. The hospital cost per stays measured by hospital charges in the dataset which is amount the hospital billed to payers include Medicare, Medicaid, private insurance and other sources for the entire hospital stay (35). The values are rounded to the nearest dollar and does not include professional fees or non-covered charges (35, 36). Due to the skewing of distribution for hospital cost per stay, we conducted the natural log of this variable. Our primary predictor was the ICD-10 code of gender dysphoria, as studied in previous literature (27). In addition, we adjusted for various patients and hospital confounders. Patient characteristics included age, race (White, Black, Hispanic, Asian or Pacific Islander, and Other), annual median household income (quartile base), primary payer (Medicare, Medicaid, Self-Pay/No Charge, Other, and Private insurance), and the severity of illness (No/Minor, Moderate, Major, Extreme comorbidity or complications). Hospital characteristics include bed size (Small, Medium, Large), ownership (Government, Private-non-profit, Private-invest-own), Location/teaching status of the hospital (Rural, Urban nonteaching, Urban teaching), and region (Northeast, Midwest, South, West).

### 2.3 Statistical analysis

Sampling weights were used for all statistical analyses to represent nationwide patients with depression. First, we examined the characteristics of the final dataset. The patient characteristics were presented as weighted frequency (percentage) or means (SD). Then we investigated the temporal trend of patients and hospital cost per stay by gender dysphoria diagnosis. Next, we explored how gender dysphoria status was associated with hospital cost per stay using the multivariate survey linear regression analysis. We also ran the models with race and census division variables to determine more specific racial and regional variances. All analyses were conducted using SAS statistical software (version 9.4; SAS Institute Inc., Cary, NC, USA). All statistical tests were two-sided and statistical significance was determined at a *p*-value <0.05.

## 3 Results

### 3.1 Patient/hospital characteristics and descriptive statistics

378,552 inpatients with depression were identified in the 2016–2019 NIS data (weighted *n* = 1,892,760, Table 1). Among them, a weighted *n* of 14,145 (0.7%) patients with gender dysphoria were hospitalized for depression. The patient and hospital demographics are presented in Table 1. Table 2 shows temporal trends for nationwide depression inpatients with/without gender dysphoria and their hospital cost per stay during 2016–2019. Our results indicate that rates of gender dysphoria among depression inpatients are increased over time, and those patients are then had higher hospital cost per stay than patients without gender dysphoria.

**Table 1:** General characteristics of sample.

Variables	Total	
	*N*	%
*N*	378,552	
Weighted *N* [National Estimates]	1,892,760	
Gender dysphoria		
No	1,878,615	99.3%
Yes	14,145	0.7%
Race		
White	1,339,330	70.8%
Black	265,510	14.0%
Hispanic	186,430	9.8%
Asian or Pacific Islander	28,100	1.5%
Other	73,390	3.9%
Age*	37.86	18.83
Sex assigned at birth		
Male	841,540	44.5%
Female	1,051,220	55.5%
Median household income		
0-25th percentile	620,085	32.8%
26th to 50th percentile	509,790	26.9%
51st to 75th percentile	432,200	22.8%
76th to 100th percentile	330,685	17.5%
Primary payer		
Medicare	348,700	18.4%
Medicaid	651,350	34.4%
Private insurance	641,170	33.9%
Self-pay	139,905	7.4%
No charge	13,420	0.7%
Other	98,215	5.2%
Severity		
No/Minor comorbidity or complications	776,795	41.0%
Moderate comorbidity or complications	1,003,240	53.0%
Major comorbidity or complications	93,470	4.9%
Extreme comorbidity or complications	19,255	1.0%
Bedsize of hospital		
Small	436,830	23.1%
Medium	528,350	27.9%
Large	927,580	49.0%
Ownership of hospital		
Government, nonfederal	204,575	10.8%
Private, non-profit	1,364,840	72.1%
Private, invest-own	323,345	17.1%
Location/teaching status of the hospital		
Rural	190,295	10.1%
Urban nonteaching	430,565	22.7%
Urban teaching	1,271,900	67.2%
Region of hospital		
Northeast	383,385	20.3%
Midwest	568,420	30.0%
South	734,285	38.8%
West	206,670	10.9%

*Mean/SD (Standard Deviation).

**Table 2:** The temporal trend for the number of nationwide depression inpatients with/without gender dysphoria and their hospital cost per stay.

Variables	2016	2017	2018	2019
*N*	90,712	97,001	96,602	94,237
Weighted *N* [National Estimates]	453,559	485,005	483,010	471,185
Gender dysphoria				
No	451,589	482,085	478,950	465,990
Yes	1,970	2,920	4,060	5,195
% of Yes	0.44%	0.61%	0.85%	1.11%
Hospital cost per stay for depression inpatients (USD)	19,374	20,147	20,815	22,166
By gender dysphoria (USD)				
No	19,361	20,131	20,797	22,151
Yes	22,497	22,753	22,960	23,474

### 3.2 Association of race and region with hospital cost per stay and length of stay

Table 3 shows the associations of gender dysphoria status with hospital cost per stay among depression inpatients. In this study, we found a statistically significant healthcare utilization disparity among hospitalized for depression patients with gender dysphoria. Patients with gender dysphoria had approximately 20.0% higher hospital cost per stay than patients without gender dysphoria. Further, higher hospital cost per stay were found in patients of minority groups, females assigned at birth, older, and from specific hospital characteristics and regions.

**Table 3:** Results of survey regression models: how gender dysphoria & hospital cost per stay are associated among depression inpatients.

Variables	Hospital Cost per stay	
	Estimation	*p*-value
Gender dysphoria		
No	Reference	
Yes	0.200	<0.0001
Race		
White	Reference	
Black	0.007	0.047
Hispanic	0.013	0.001
Asian or Pacific Islander	0.102	<0.0001
Other	0.090	<0.0001
Age	0.003	<0.0001
Sex assigned at birth		
Male	−0.025	<0.0001
Female	Reference	
Median household income		
0-25th percentile	Reference	
26th to 50th percentile	−0.016	<0.0001
51st to 75th percentile	0.043	<0.0001
76th to 100th percentile	0.144	<0.0001
Primary payer		
Medicare	0.192	<0.0001
Medicaid	0.076	<0.0001
Private insurance	Reference	
Self-pay	−0.034	<0.0001
No charge	0.095	<0.0001
Other	−0.026	<0.0001
Severity		
No/Minor comorbidity or complications	Reference	
Moderate comorbidity or complications	0.227	<0.0001
Major comorbidity or complications	0.639	<0.0001
Extreme comorbidity or complications	1.417	<0.0001
Bedsize of hospital		
Small	Reference	
Medium	0.062	<0.0001
Large	0.172	<0.0001
Ownership of hospital		
Government, nonfederal	Reference	
Private, non-profit	−0.008	0.036
Private, invest-own	0.483	<0.0001
Location/teaching status of the hospital		
Rural	Reference	
Urban nonteaching	0.137	<0.0001
Urban teaching	0.302	<0.0001
Region of hospital		
Northeast	0.437	<0.0001
Midwest	Reference	
South	−0.011	<0.0001
West	0.286	<0.0001
Year	0.054	<0.0001

### 3.3 Sub-group models with race and specific region variables

Table 4 shows the results of the subgroup analysis. Depression inpatients with gender dysphoria were associated with higher hospital costs per stay for most racial groups. This trend also holds in the model where the region variable was replaced with a census division variable. Again, in most census divisions, depression inpatients with gender dysphoria were associated with higher hospital costs per stay.

**Table 4:** Results of survey regression models: sub-groups analysis by race and region.

Variables	Hospital Cost per stay	
	Estimation	*p*-value
Race		
White	0.205	<0.0001
Black	0.221	<0.0001
Hispanic	0.219	<0.0001
Asian or Pacific Islander	0.204	0.042
Other	0.031	0.653
Census division of hospital		
New England	0.091	0.052
Middle Atlantic	0.241	<0.0001
East North Central	0.185	<0.0001
West North Central	0.254	<0.0001
South Atlantic	0.183	<0.0001
East South Central	0.311	<0.0001
West South Central	0.220	<0.0001
Mountain	0.087	0.089
Pacific	0.168	<0.0001

All other variables were adjusted.

## 4 Discussion

Across the study periods, the proportion of patients with gender dysphoria hospitalized for depression increased. The growth in the number of individuals with gender dysphoria is consistent with previous literature, which describes how growing acceptance and discussion about genders has allowed patients to identify their gender openly (9). Social and cultural acceptance is vital, but medical support and access are still lacking (19, 22, 24). Overall cost was steady for patients with depression and gender dysphoria and patients with only depression. However, patients with depression and gender dysphoria were charged significantly more than those without. Patients with gender dysphoria are at an increased risk for mental health problems (2, 12, 16), which could account for their increased hospital cost per stay due to increased complexity. While the increased expense is explainable, it is still a relevant disparity because the increased healthcare cost may become a barrier to seeking transition treatment, which may improve mental health (6, 33). Puckett’s previous study demonstrated that financial barriers, insurance coverage, and provider bias are common obstacles to gender-affirming care (34). While individuals with gender dysphoria and depression may benefit from gender-affirming therapy, they may be financially restricted (20, 29, 31, 33, 37). Economic disparities and associated healthcare costs, like the hospital cost per stay identified here, lock them in a vicious cycle (20, 31).

Additionally, individuals in the median 26th-50th percentile for household income had lower hospital costs per stay. Depending on the year, based on the NIS, the median household income in this percentile range was between $43,000 and $60,999 (38). In contrast, the 51st-75th and 76th to 100th percentile, representing higher median household income range (38), are significantly and positively associated with hospital costs per stay. Therefore, higher incomes pay more for gender dysphoria and depression hospitalization, which could signal increased healthcare utilization. Their increased healthcare utilization infers greater access to care for their gender dysphoria and depression. Slaughter and colleagues also found that individuals with gender dysphoria were more likely to undergo gender-affirming surgeries if they were from higher socio-economic groups or had private insurance (19). The lower charges in our study for the 26th-50th percentile median household income may indicate decreased healthcare utilization, possibly due to reduced access. The 2015 US Transgender Survey found that transgender individuals have high rates of poverty, double the national average, and unemployment (9). With such financial restrictions, individuals with gender dysphoria and depression are further burdened by healthcare costs. Patients in this income bracket may not be able to afford hospitalization charges and could avoid seeking treatment for financial reasons, leading to decreased costs from decreased utilization.

Our study had similar results to Slaughter’s research, which found higher rates of gender-affirming surgery with private insurance (19). Our results demonstrate that self-pay or “other” payment methods had lower hospital costs per stay compared to private insurance. In contrast, Medicare had a significantly higher association with hospital cost per stay, possibly due to improved coverage for gender-affirming surgery and hormone therapy since 2014 (39). However, despite improved coverage, hormone therapies remain expensive, and prior authorizations and formulary restrictions mitigate advances (37). Since finances and insurance have been shown to be obstacles to care for patients with gender dysphoria and depression is highest in low-income levels (18), our findings support improving insurance coverage and access to gender-affirming treatments and psychological therapy to mitigate costs.

Hospital cost per stay also differed based on the sex assigned at birth. People assigned males at birth were charged significantly less than females assigned at birth. Previous literature found that males assigned at birth have higher rates of gender-affirming surgery than females assigned at birth (19). Gender-affirming therapies have been known to improve the mental health of patients with gender dysphoria (6, 31–33). In addition, females assigned at birth tend to have a higher proportion of depression than males assigned at birth (18). Females assigned birth historically earn less than their male counterparts (38). Ergo, when combining the increased risk for depression, lower rates of gender-affirming surgery, and decreased income, individuals who are female assigned at birth are disproportionally marginalized. Encumbered by financial disparities and higher rates of depression that, for economic reasons, are less likely to be alleviated by gender-affirming therapies, they are then charged more for associated hospitalizations (6, 20, 29, 31–33, 37).

To understand how specific populations with gender dysphoria and depression access healthcare, we ran the sub-group analysis by race and region (Table 4). While White, Black, and Hispanic individuals were all significantly associated with higher hospital costs per stay for gender dysphoria and depression, Black and Hispanic individuals were charged the most. Literature on racial differences in gender dysphoria is limited (24–27) but shows similar trends. Since gender affirmation therapies can be helpful for the mental health of those who want it (6, 31–33), the decreased diagnosis for gender dysphoria or hormonal treatment among racial and ethnic minorities is of grave concern. Racial and ethnic differences in mental health are well documented and could be exacerbated by added bias for gender dysphoria and depression (11, 12, 16, 40, 41). The increased hospital cost per stay for Black and Hispanic patients are an excessive burden considering that both groups earn less than White individuals (38). Previous literature has also posited that racial minorities and low-income groups have unequal access to care (16, 22, 24–28). Racial and ethnic minorities who are transgender also have unemployment rates higher than the national average, negative experiences with healthcare providers, and are three times more likely than the national average to live in poverty (9). Other studies have shown that being of a racial or ethnic minority is also associated with being more likely to be refused care (29). Hence, our results support further research on racial and ethnic individuals’ experience with gender dysphoria and discussions on cultural considerations for improved care and access. Culturally competent care may be able to play a dual role in affirming patients’ identities and recognizing their backgrounds.

Almost all census divisions were significantly associated with increased hospital costs per stay, but the highest was in East South Central, West North Central, Middle Atlantic, and West South Central. Our first model found that the South was the only region significantly and negatively associated with hospital cost per stay. Due to state differences in insurance coverage for hormonal therapy and gender-affirming surgery, there is a wide variation in the cost for individual patients (20). Southern states are among the highest in transgender care refusal (29), and Arkansas (located in West South Central) in 2021 banned gender-affirming care for minors (20). Of the census divisions identified as having the highest hospital cost per stay for gender dysphoria and depression, only the Middle Atlantic and Minnesota in the West North Central have laws preventing insurance companies from discriminating against transgender groups (20, 42). As political climates discriminate against the LGBT community, social acceptance decreases, and the South has the lowest approval in the US; in the Midwest (which covers the West North Central), LGBT individuals have significantly reduced incomes (43). With LGBT inclusivity tied to economic development, excluding patients with gender dysphoria has broader critical economic outcomes (44). For example, workplace discrimination and health disparities can lead to decreased productivity (44). Such discrimination can add to individuals’ depression and anxiety, leading to more complex and severe cases that are more expensive to treat. Our study found a significantly increasing association with hospital cost per stay as severity increased with the highest costs for extreme comorbidities or complications. Individuals in East South Central, West South Central, and West North Central then remain imprisoned by growing depression and lack of treatment for gender dysphoria, financial barriers to care, and social stigma that keep them from a better quality of life. The high hospital cost per stays identified here exemplifies how difficult it is for them to seek care. In the Middle Atlantic, where there is more social and political support for the LGBT community (20, 42, 43), the high hospital cost per stay indicates that more work is needed to achieve health equity.

## 5 Limitations

Though this study explores critical differences in hospital cost per stay for patients with depression and gender dysphoria, its application has some limitations. First, the dataset does not include patient perspectives on healthcare access or cost, which could be used to identify relevant biases. Also, the NIS Dataset uses ICD-10 codes for major depressive disorder and gender dysphoria, limiting patient selection. This study focused on depression, which patients with gender dysphoria are at increased risk for, but other mental health conditions like anxiety could be explored. Future studies could focus on other mental health conditions. The dataset also does not have information on if patients received or were interested in any gender-affirming therapy. Lastly, no clinical information on case severity was included, limiting real-life interpretations. Further research is required to determine the number of individuals with gender dysphoria and their access to healthcare. Still, this study illuminated unique factors related to increased hospital cost per stay for depression in patients with gender dysphoria. Such information is vital to promoting appropriate care to prevent expensive hospitalizations and further marginalization.

## 6 Conclusion

Across sub-groups, we found evidence of disproportionately higher hospital costs per stay. Following a worrying trend in previous literature, we identified that certain genders and racial and ethnic minorities are at an increased risk of health disparities. Further, regional differences in insurance coverage and political protections may adversely affect patients’ mental health and keep them from advancing in gender affirmation therapies. Patients charged more for hospitalization can be prevented from accessing future treatments due to financial reasons and are burdened by unemployment, poverty, and discrimination risks. Patients unable to afford gender-affirming therapies risk inadequate management of their gender dysphoria and mental health, potentially adding complexity to their cases. Increased comorbidities and complexity were associated with hospital costs. Consequently, some patients with gender dysphoria remain unable to afford supportive therapies and may be overburdened by the resultant hospitalization price for depression. To reach health equity, the US must assure insurance coverage for gender-affirmation treatments, codify legal protections against discrimination, and increase medical provider access and education.

## Data availability statement

The original contributions presented in the study are included in the article/supplementary material, further inquiries can be directed to the corresponding author.

## Ethics statement

The studies involving humans were approved for a waiver from the Institutional Review Board, Soonchunhyang University (202203-SB-027). The studies were conducted in accordance with the local legislation and institutional requirements. Written informed consent for participation was not required from the participants or the participants’ legal guardians/next of kin in accordance with the national legislation and institutional requirements.

## Author contributions

SK: Conceptualization, Data curation, Funding acquisition, Methodology, Validation, Writing – original draft, Writing – review & editing. MM: Investigation, Resources, Visualization, Writing – original draft, Writing – review & editing. J-HP: Conceptualization, Resources, Visualization, Writing – review & editing, Writing – original draft. N-EC: Resources, Visualization, Writing – review & editing, Writing – original draft. JC: Conceptualization, Data curation, Resources, Supervision, Writing – original draft, Writing – review & editing.
